# Role of daytime variation in pharmaceutical effects of sufentanil, dezocine, and tramadol: A matched observational study

**DOI:** 10.3389/fphar.2022.993506

**Published:** 2022-09-16

**Authors:** Wanxia Gan, Xinqing Yang, Jie Chen, Hongyao Lyu, Ai Yan, Guizhen Chen, Shiqi Li, Yamei Zhang, Ling Dan, He Huang, Guangyou Duan

**Affiliations:** ^1^ Department of Anesthesiology, The Second Affiliated Hospital, Chongqing Medical University, Chongqing, China; ^2^ Department of Preventive Medicine, West China School of Public Health, Sichuan University, Chengdu, Sichuan, China

**Keywords:** daytime variation, pharmaceutical effect, tramadol, sufentanil, dezocine

## Abstract

The role of daytime variation in the comprehensive pharmaceutical effects of commonly used opioid analgesics in clinical setting remains unclear. This study aimed to explore the differences in daytime variation among elective surgery patients who were scheduled to receive preemptive analgesia with equivalent doses of sufentanil, dezocine, and tramadol in the morning and afternoon. The analgesic effect was assessed by changes in the pressure pain threshold before and after intravenous administration of sufentanil, dezocine, and tramadol. Respiratory effects were evaluated using pulse oximetry, electrical impedance tomography, and arterial blood gas analysis. Other side effects, including nausea, sedation, and dizziness, were also recorded, and blood concentration was measured. The results showed that the analgesic effects of sufentanil, dezocine, and tramadol were significantly better in the morning than in afternoon. In the afternoon, sufentanil had a stronger sedative effect, whereas dezocine had a stronger inhibitory respiratory effect. The incidence of nausea was higher in the morning with tramadol. Additionally, significant differences in different side effects were observed among three opioids. Our results suggest that the clinical use of these three opioids necessitates the formulation of individualized treatment plans, accounting for different administration times, to achieve maximum analgesic effect with minimal side effects.

## Introduction

Every year, more than 312.9 million operations are performed worldwide (Weiser et al., 2008; Weiser et al., 2016; Song and Zhu, 2021). Opioids are among the most important and commonly used intravenous analgesics for perioperative analgesia. However, opioid analgesics are often accompanied by various side effects, including respiratory depression, sedation, dizziness, and nausea (Nakatani, 2017; Baldo, 2021). Excessive drug use can lead to an increased incidence of complications, while inadequate drug use can lead to insufficient analgesic effect, which significantly affects perioperative recovery. Previous studies regarding the influence of time variation on pharmaceutical efficacy have indicated that the selection of drug treatment at different time points is important for achieving the best efficacy with minimum dose and minimal side effects (Lemmer, 1995; Warltier David et al., 2004; Saleem et al., 2019; Zaki et al., 2019). Exploring the role of time variation in the pharmaceutical effect of analgesics is expected to be beneficial for perioperative patients.

The diurnal effects of opioid efficacy and treatment sensitivity have been observed in previous studies. For example, it was found that the analgesic effect of morphine had circadian rhythm changes in mice, and that it was strongest at night (Yoshida et al., 2003; Yu et al., 2015). In addition, studies in healthy volunteers have found that oral tramadol exerts a stronger analgesic effect at night (Hummel et al., 1995). However, these studies were confined to animals or healthy volunteers, and the time points were mainly focused on morning and night hours. Additionally, the side effects of these opioids have been less explored. In clinical practice, most surgeries and therapeutic treatments are performed during the daytime (Cifarelli et al., 2021); therefore, it is more important to explore the potential differences in the clinical and side effects of analgesics between morning and afternoon hours.

Currently, in clinical practice, the most commonly used opioid analgesics are pure µ-receptor agonists, such as sufentanil and norepinephrine, 5-hydroxytryptamine reuptake inhibitors, such as tramadol, and k-receptor agonists and μ-receptor antagonists, such as dezocine. These three types of opioid analgesics have different mechanisms of action (Bravo et al., 2017; Ye et al., 2021). At present, the effects of daytime variations on the pharmaceutical effects of these three opioids remain unclear. In addition, direct clinical evidence is lacking to comprehensively evaluate the clinical effect of these three types of opioid analgesics. Based on the above information, this study aimed to comprehensively evaluate and compare the pharmaceutical effects of these analgesics by experimental pain measurement, real-time continuous pulmonary ventilation monitoring, and cardiovascular parameter monitoring.

## Materials and methods

### Subjects

The study was designed as a prospective matched observational study to determine the role of daytime variation in the pharmaceutical effects of three types of commonly used opioid analgesics with different mechanisms. The research protocol was in accordance with the tenets of Declaration of Helsinki and approved by the Medical Ethics Committee of the Second Affiliated Hospital of Chongqing Medical University and registered at www.chictr.org.cn [Registration number: ChiCTR2100044369 (Tramodol), ChiCTR2100050360 (Dezocine) and ChiCTR2100053467 (Sufentanil)]. All subjects signed a written informed consent form prior to inclusion in the study. All study data can be obtained by email from the corresponding author on reasonable request.

A total of 300 patients who required pre-analgesia before their operations at the Second Affiliated Hospital of Chongqing Medical University from March 2021 to May 2022 were included. In clinical practice most of the surgeries and treatments were performed in day-time (from 07:00 to 17:00), and 12:00 is commonly used as the cutoff time point between morning and afternoon, thus in this study subjects were grouped as “07:00 to 12:00” and “12:00–17:00”. Considering these three opioids are wildly used clinically, we selected tramadol, dezocine and sufentanil as investigative analgesics. Patients in this study were sequentially enrolled in the tramadol (*n* = 100), dezocine (*n* = 100), and sufentanil (*n* = 100) experiments. Patients in each experiment were divided into the morning (M group, 07:00 to 12:00, *n* = 50) and afternoon groups (A group, 12:00 to 17:00, *n* = 50) according to administration time.

Inclusion criteria were: 18–65 years old; 18.5 ≤ Body Mass Index (BMI) ≤ 28; American Society of Anesthesiologists grade I-II; patients undergoing elective surgery. Exclusion criteria were: long-term use of analgesics and psychotropic drugs [including opioids, nonsteroidal anti-inflammatory drugs (NSAIDs), sedatives, and antidepressants]; alcohol abuse, liver and renal dysfunction; history of opioid allergy; use of sedatives and antiemetic drugs within 24 h; use of monoamine oxidase inhibitors or antidepressants within 15 days; high risk of emergency, satiety, and reflux aspiration; history of epilepsy; history of severe chronic obstructive pulmonary disease; severe or uncontrolled bronchial asthma; pulmonary infections; severe heart disease; pregnant or lactating women; and an inability to cooperate with the study for any reason.

### Study protocol

After entering the operating room, the subjects’ demographic and physiological data at baseline and the Huaxi Emotional Index were collected (Wang et al., 2017). Electrocardiography, blood pressure, arterial blood gas analysis (ABGs), SPO_2_ and Modified Observer’s Assessment of Alertness/Sedation (MOAA/S) were monitored before drug administration. Baseline pressure pain threshold (PPT) was measured. Electrical Impedance Tomography (EIT) monitoring was performed to record the baseline respiratory rate and change in end-expiratory lung impedance (Δ EELI). During the post-dosing test, subjects were treated with deep breathing if SPO_2_ was lower than 85% and oxygen inhalation *via* mask if SPO_2_ was lower than 80%.

**FIGURE 1:**
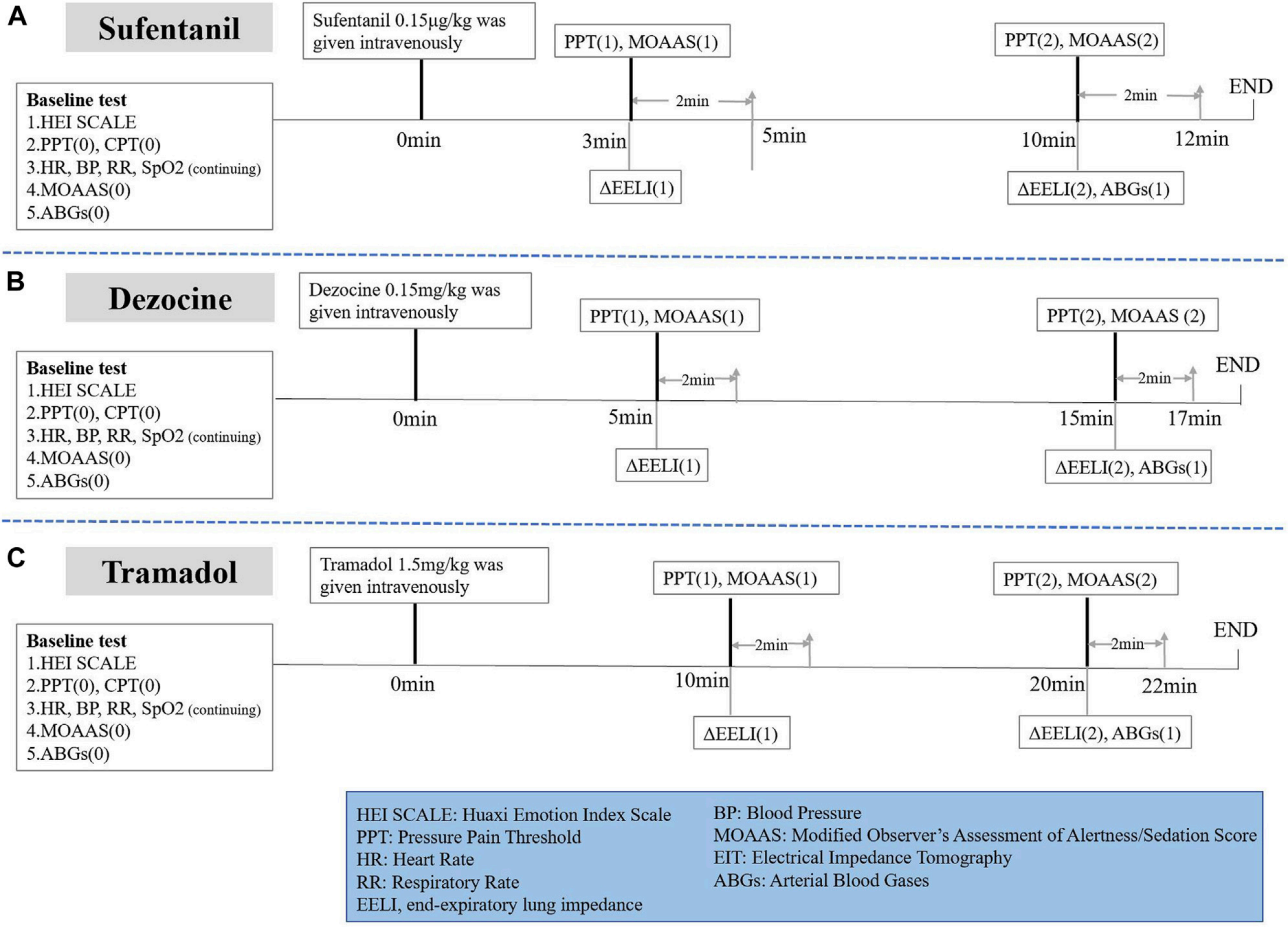
Study protocol for different analgesics.

As shown in Figure 1A, subjects in the sufentanil group were given sufentanil 0.15 μg/kg intravenously over 1 min. PPT and MOAA/S scores were tested at 3 min (onset time of analgesic effect) and 10 min (time when the peak drug concentration was reached) after administration. ABGs were performed again 10 min after administration. We recorded ΔEELI at 3 and 10 min after administration through EIT monitoring.

In addition, as shown in Figure 1B, subjects in the dezocine group were given dezocine 0.15 mg/kg intravenously over 1 min. PPT and MOAA/S scores were tested at 5 min (onset time of analgesic effect) and 15 min (time when the peak drug concentration was reached) after administration. We performed ABGs again 15 min after administration. We recorded ΔEELI at 5 and 15 min after administration using EIT monitoring.

Subjects in the tramadol group were given tramadol 1.5 mg/kg intravenously over 1 min. PPT and MOAA/S scores were tested at 10 min (onset time of analgesic effect) and 20 min (when the peak drug concentration was reached) after administration. ABGs were performed again 20 min after administration. ΔEELI at 10 and 20 min after administration was recorded using EIT monitoring (Figure 1C).

### Analgesic effect measurement

Quantitative sensory tests including PPT have been widely used to assess analgesic effect in many previous studies (Hermans et al., 2018; Schliessbach et al., 2018; Ho et al., 2020). Thus, in this study the primary outcome was a normalized value compared to the baseline PPT after analgesic administration. The PPT was measured using a hand-held electro-mechanical algorithm instrument (YISIDA-DS2; Hong Kong, China), which consisted of a pressure sensor with a probe surface area of 0.1 cm^2^ (Duan et al., 2014; Duan et al., 2015). The right tibialis anterior muscle, which was approximately 1–2 cm lateral to the middle tibia, was selected as the test site. Two adjacent sites at this location were marked to ensure that the investigator could repeat PPT measurements at approximately the same measurement site. The subjects lay flat on the push table in a relaxed position and were subjected to continuous pressure at approximately the same rate (0.3 kg/s) during the test. In order to avoid unnecessary tissue damage, the maximum pressure did not exceed 5 kg. When the subjects began to feel pain during the stimulation, they were asked to say “pain” and the pressure value was recorded. The average of the values at the two adjacent sites was recorded as the PPT of the subject (Duan et al., 2020).

### Respiratory effect evaluation

The secondary outcome in this study was respiratory effects after analgesic use. ABGs were performed and PaO_2_ and PaCO_2_ were recorded to assess the overall effect of analgesic use on the respiratory system. EIT represented a noninvasive bedside method of monitoring the respiratory rate and impedance changes associated with different pulmonary ventilation statuses, especially those related to regional ventilation impedance change, to reflect the regional features of the lung (Zhang et al., 2020). In this study, EIT monitoring was used to record the effects of analgesics on respiration. There is a good correlation between the change in end-expiratory lung impedance (EELI) and the change in end-expiratory lung volume (Moerer et al., 2011). The change in EELI (ΔEELI) was measured relative to baseline EELI. Desaturation and bradypnea were also recorded. Desaturation was defined as SpO_2_ < 95% during the entire procedure after analgesic administration, and bradypnea was defined as a respiratory rate < 8.

### Other outcomes

In this study, other outcomes mainly included the incidence of sedation, dizziness, nausea, and vomiting. Sedation was defined as a MOOA/S score of < 5, and all subjects were assessed twice after analgesic administration. Dizziness was evaluated by interviewing the subjects during the entire test procedure. Vomiting was recorded according to observations after analgesic administration, and nausea was assessed after the study procedure. The numeric rating scale (NRS, 0 representing none and 10 representing the worst imaginable) for nausea was recorded.

### Serum detection

Blood specimens (3 ml) were collected from the radial artery using a vacuum tube with heparin after the last pressure pain measurement in all subjects. Serum was separated and stored at −80°C. Sufentanil and tramadol concentrations were detected using an enzyme-linked immunosorbent assay (ELISA) kit. The concentration of dezocine was determined using high performance liquid chromatography (HPLC).

### Statistical analysis

This study was considered as an exploratory study to determine the role of daytime variation on the pharmaceutical effects of three types of commonly used opioid analgesics. Therefore, we primarily included the same sample size for different opioid analgesics. According to the primary outcome of this study, that is, normalized value compared to baseline PPT after analgesic administration, a power calculation showed that the current sample size for three types of opioid analgesics could reach a power > 0.8.

Statistical analysis was performed using SPSS 21.0, and two-sided *p* < 0.05 was considered statistically significant. Normally distributed quantitative data were described as mean ± standard deviation, and abnormally distributed data were presented as median (interquartile range). Qualitative data are presented as numbers (percentages). Independent sample T test, non-parametric Mann–Whitney test, or two-way repeated measures analysis of variance (ANOVA) were used to compare the morning and afternoon groups according to whether the data were normally distributed. Chi-square test or Fisher’s exact test was used to compare the incidence between the morning and afternoon groups.

Because the analgesic effect was better in the morning group, we comprehensively compared the different clinical effects of the three types of analgesics. Differences in various normally distributed quantitative parameters among the three groups were analyzed using ANOVA and post-hoc multiple comparisons with Bonferroni correction. The Kruskal–Wallis test was performed to compare the abnormally distributed data among the three groups and post-hoc multiple comparisons with Bonferroni corrections. Chi-square test or Fisher’s exact test was used for comparison of incidence between the three groups, and multiple comparisons between different analgesics were also corrected by the Bonferroni method.

## Results

**FIGURE 2:**
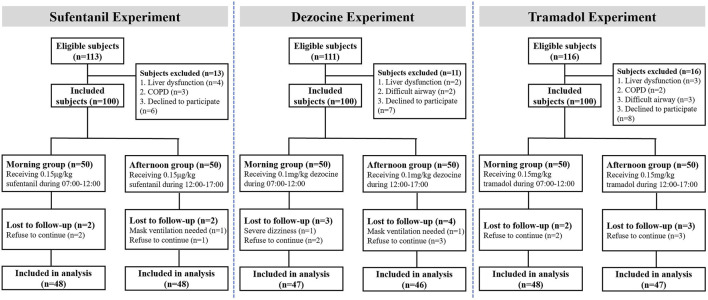
Flow chart of the subjects’ inclusion in the study.

As shown in Figure 2, in the study 3 subjects in sufentanil experiment, seven subjects in the dezocine experiment and 5 subjects in the tramadol experiment were lost to follow-up. Thus, 96 patients receiving sufentanil (48 in each morning and afternoon group), 93 patients receiving dezocine (47 in the morning group and 46 in the afternoon group), and 95 patients receiving tramadol (48 in the morning group and 47 in the afternoon group) were included in the final analysis. The demographic and baseline characteristics of all included subjects in the morning and afternoon groups are listed in Table 1, and no significant differences were found in the three experiments.

**TABLE 1:** Demographic and baseline characteristics for all included subjects in the morning and afternoon groups.

	Sufentanil			Dezocine			Tramadol		
	Morning (*n* = 48)	Afternoon (*n* = 48)	*P*	Morning (*n* = 47)	Afternoon (*n* = 46)	*P*	Morning (*n* = 48)	Afternoon (*n* = 47)	*P*
Age (year)	44.5 ± 10.6	40.8 ± 11.7	0.107	41.6 ± 9.4	44.4 ± 11.2	0.188	42.4 ± 10.9	41.6 ± 10.5	0.731
Sex (male)	14 (29.2%)	18 (37.5%)	0.386	9 (19.1%)	13 (28.3%)	0.301	10 (20.8%)	7 (14.9%)	0.450
Weight (kg)	62 ± 9	62 ± 10	0.356	62 ± 9	61 ± 9	0.788	60 ± 8	60 ± 10	0.792
Height (cm)	161 ± 7	162 ± 9	0.965	161 ± 8	161 ± 8	0.874	161 ± 6	159 ± 6	0.127
BMI (kg/m^2^)	23.9 ± 2.8	23.4 ± 2.6	0.389	23.8 ± 2.8	23.5 ± 2.9	0.646	23.0 ± 2.6	23.8 ± 3.1	0.204
HEI	4.0 (1.0–8.2)	3.0 (1.0–6.0)	0.326	4.0 (2.0–7.0)	4.0 (1.0–5.3)	0.341	5.0 (3.0–7.0)	4.5 (1.8–7.3)	0.593
MAP (mmHg)	99 ± 13	96 ± 10	0.227	97 ± 10	99 ± 10	0.504	94 ± 11	96 ± 12	0.369
HR (bpm)	76 ± 11	74 ± 11	0.369	76 ± 12	73 ± 10	0.283	75 ± 8	75 ± 11	0.689
RR (bpm)	15.3 ± 3.0	15.8 ± 3.2	0.394	15.6 ± 3.7	16.8 ± 3.5	0.105	16.5 ± 4.4	16.8 ± 4.2	0.745
S_P_O_2_ (%)	98.5 ± 1.5	99.0 ± 1.2	0.436	98.6 ± 1.5	98.7 ± 1.6	0.802	98.6 ± 1.4	98.4 ± 1.8	0.460

Data are presented as mean ± standard deviation, number (percentage) or median (interquartile range); BMI, body max index; HEI, huaxi emotion index scale; MAP, mean arterial pressure; HR, heart rate; RR, mean arterial pressure; PPT, pressure pain threshold.

**FIGURE 3:**
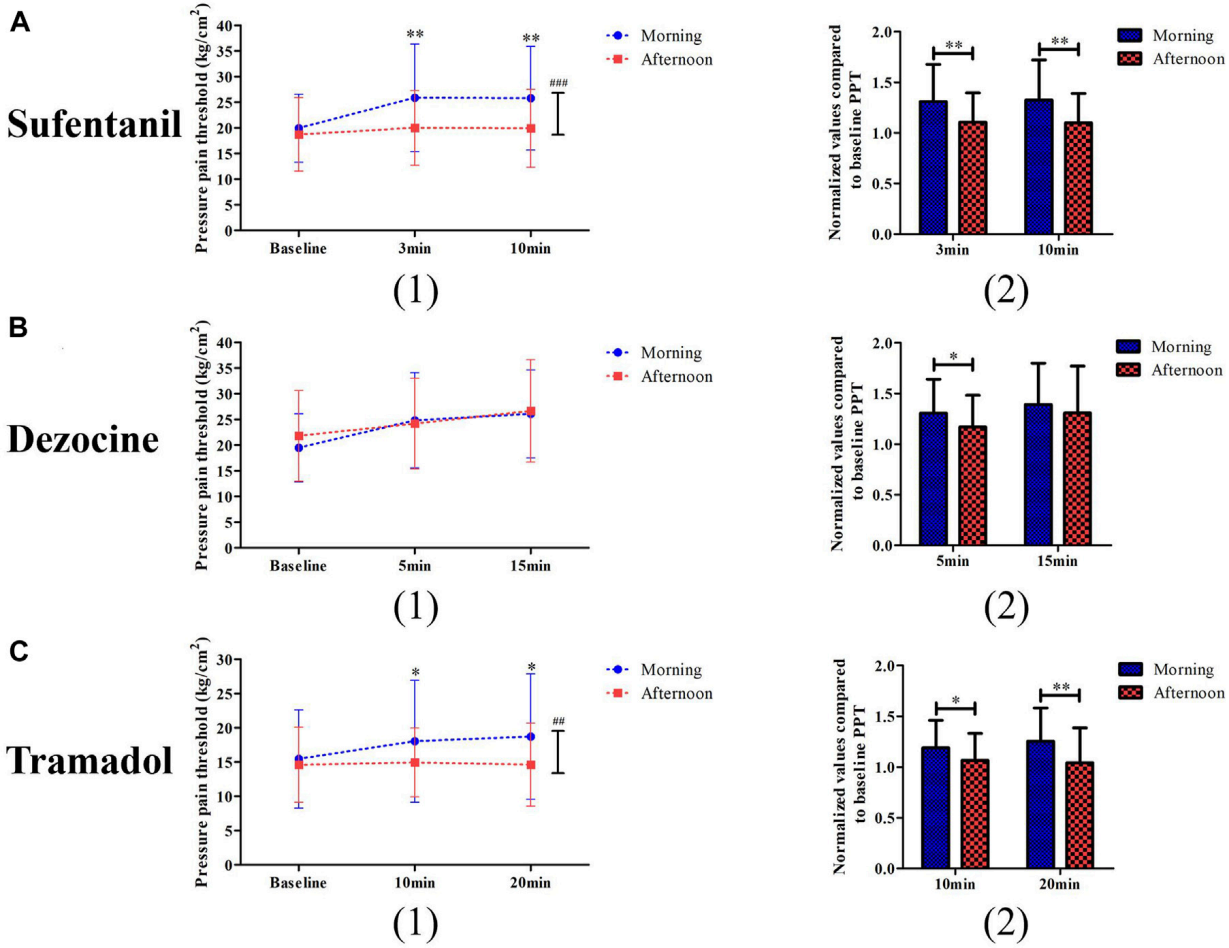
Comparisons of pressure pain threshold (1) and its changed levels (2) between the morning group and afternoon group for patients receiving sufentanil treatment **(A)**, dezocine treatment **(B)** and tramadol treatment **(C)**. (*p < 0.05 and **p < 0.01 compared with the afternoon group; ^##^ *p* < 0.01 and ^###^ *p* < 0.001 represents the time and group effects for two way repeat ANOVA analysis).

Two-way repeated ANOVA analysis showed that the interactive effects of time and group for pressure pain threshold at different time points for sufentanil (*p* < 0.001) and tramadol (*p* = 0.001) were statistically significant, but not for dezocine (*p* = 0.062, Figure 3). In the sufentanil experiment, both of the normalized values compared to baseline PPT at 3 min (1.31 ± 0.37 vs. 1.11 ± 0.29, *p* = 0.003) and 10 min (1.32 ± 0.39 vs. 1.10 ± 0.29, *p* = 0.002) in the morning group are higher than those in the afternoon group (Figure 2A). In the dezocine experiment, as shown in Figure 3B the normalized values compared to baseline PPT at 5 min (1.31 ± 0.34 vs. 1.17 ± 0.31, *p* = 0.046) in the morning group is higher than that in the afternoon group, and no difference was found at 15 min (1.39 ± 0.41 vs. 1.31 ± 0.46, *p* = 0.363). And in the tramadol experiment, both of the normalized values compared to baseline PPT at 10 min (1.19 ± 0.27 vs. 1.07 ± 0.27, *p* = 0.028) and 20 min (1.25 ± 0.33 vs. 1.04 ± 0.34, *p* = 0.003) in the morning group are higher than those in the afternoon group (Figure 3C).

**FIGURE 4:**
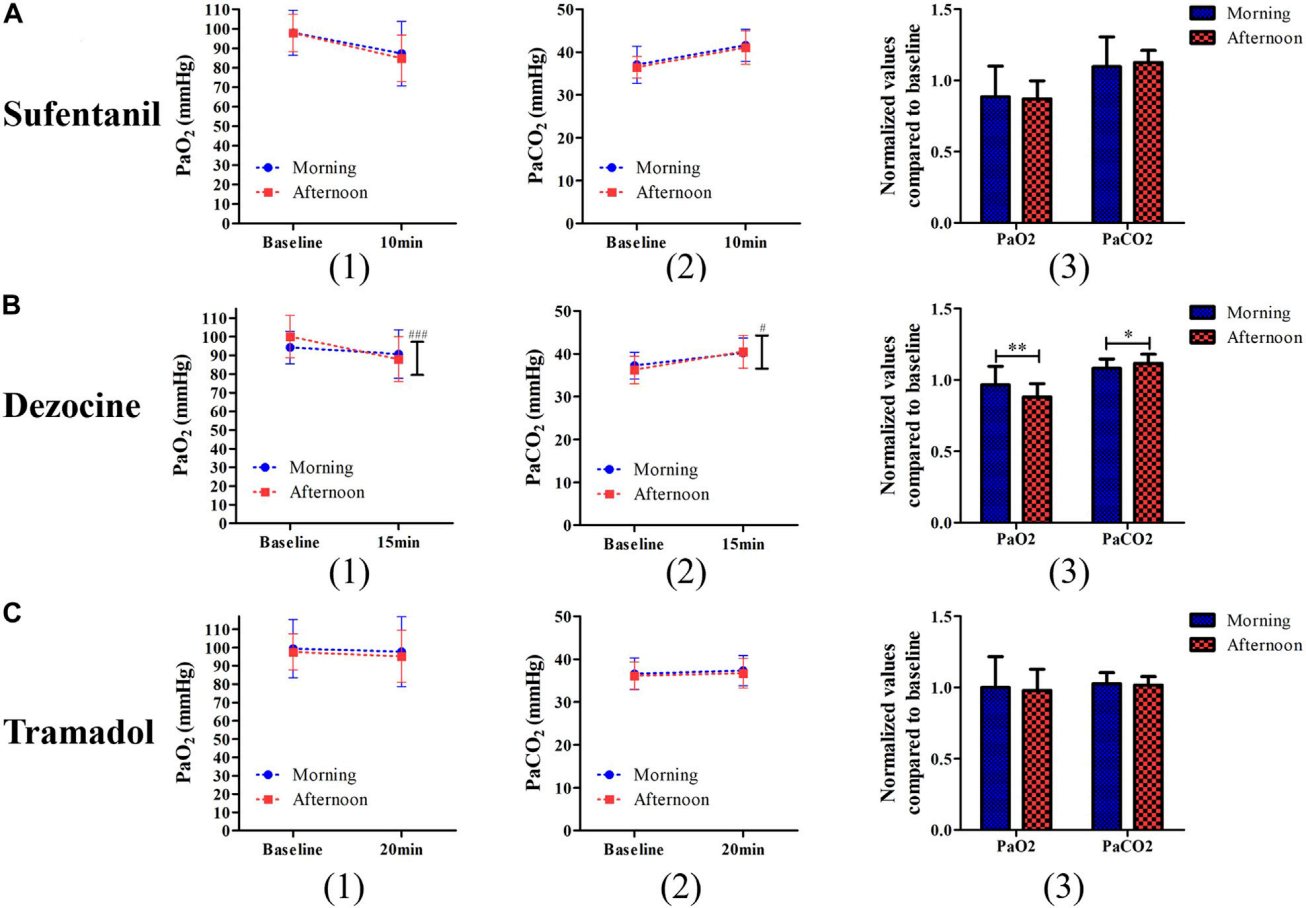
Comparisons of PaO_2_ (1) and PaCO_2_ (2) and their changed levels (3) after test procedure between the morning group and afternoon group for patients receiving sufentanil treatment **(A)**, dezocine treatment **(B)** and tramadol treatment **(C)**. (*p < 0.05 and **p < 0.01 compared with the afternoon group; ^#^ *p* < 0.05 and ^###^ *p* < 0.001 represents the time and group effects for two way repeat analysis of variance).

No significant difference was found in blood pressure or heart rate during the study procedure for the different analgesic experiments (Supplementary Figure S1). In the two-way repeated analysis of PaO_2_ (*p* < 0.001) and PaCO_2_ (*p* = 0.014), the interactive effects of time and group for dezocine were statistically significant, and no significant difference was found in the sufentanil and tramadol experiments (Figure 4). In the dezocine experiment, both normalized values compared to baseline PaO_2_ (1.31 ± 0.37 vs. 1.11 ± 0.29, *p* = 0.003) and PaCO_2_ (1.32 ± 0.39 vs. 1.10 ± 0.29, *p* = 0.002) were higher in the morning group than in the afternoon group. The other outcomes of the morning and afternoon groups are listed in Table 2. In the sufentanil experiment, the incidence of sedation (52.1% vs. 22.9%, *p* = 0.003) 10 min after analgesic use in the afternoon group was significantly higher than in the morning group. In addition, the incidence of nausea (50.0% vs. 29.8%, *p* = 0.043) during the study procedure was significantly higher in the morning group than in the afternoon group of the tramadol experiment.

**TABLE 2:** Comparisons of side effects between the morning and afternoon groups after different analgesic using.

	Sufentanil			Dezocine			Tramadol		
	Morning (*n* = 48)	Afternoon (*n* = 48)	*P*	Morning (*n* = 47)	Afternoon (*n* = 46)	*P*	Morning (*n* = 48)	Afternoon (*n* = 47)	*P*
Sedation at T1	23 (47.9%)	27 (56.3%)	0.414	18 (38.3%)	13 (28.3%)	0.305	0 (0.0%)	2 (4.3%)	0.466
Sedation at T2	11 (22.9%)	25 (52.1%)	0.003	17 (36.2%)	12 (26.1%)	0.294	2 (4.2%)	1 (2.1%)	0.566
Desaturation	41 (85.4%)	34 (70.8%)	0.084	29 (61.7%)	25 (54.3%)	0.472	6 (12.5%)	9 (19.1%)	0.374
Bradypnea	26 (54.2%)	30 (62.5%)	0.408	30 (63.8%)	24 (52.2%)	0.255	26 (54.2%)	17 (36.2%)	0.078
ΔEELI at T1	−0.15 (−0.29∼−0.02)	−0.19 (−0.32–0.12)	0.919	−0.25 (−0.40∼−0.08)	−0.24 (−0.48∼−0.09)	0.677	−0.20 (−0.39–0.36)	−0.09 (−0.50–0.18)	0.631
ΔEELI at T2	−0.32 (−0.64∼−0.08)	−0.26 (−0.67∼−0.09)	0.345	−0.42 (−0.72∼−0.13)	−0.43 (−0.82–0.20)	0.908	−0.30 (−0.69–0.06)	−0.31 (−0.92–0.13)	0.748
Dizziness	48 (100%)	46 (95.8%)	0.475	46 (97.9%)	42 (91.3%)	0.345	26 (54.2%)	18 (38.3%)	0.121
Nausea	12 (25.0%)	6 (12.5%)	0.117	4 (8.5%)	2 (4.3%)	0.693	24 (50.0%)	14 (29.8%)	0.043
NRS of nausea	0.0 (0.0–0.8)	0.0 (0.0–0.0)	0.139	0.0 (0.0–0.0)	0.0 (0.0–0.00)	0.406	0.5 (0.0–3.8)	0.0 (0.0–1.0)	0.033

Data are presented as mean ± standard deviation, number (percentage) or median (interquartile range); T1, time point for first pain measurement; T2, time point for second pain measurement; ΔEELI, changed end-expiratory lung impedance; NRS, number rating scale.

The blood detection results between the morning and afternoon groups after different analgesic uses are listed in Table 3. In the sufentanil experiment, no significant difference was found in sufentanil concentration between the morning and afternoon groups. In the tramadol experiment, tramadol concentration (658 ± 241 vs. 0.915 ± 352 ng/ml, *p* < 0.001) in the morning group was lower than that in the afternoon group. Finally, no significant differences were observed in the dezocine experiment.

**TABLE 3:** Comparisons of blood detection results between the morning and afternoon groups after different analgesic using.

	Morning	Afternoon	*P*
Sufentanil concentration(pg/ml)	1.0 (1.0–2.9)	1.0 (1.0–3.0)	0.820
Dezocine concentration(ng/ml)	10.7 ± 2.2	11.5 ± 3.6	0.199
Tramadol concentration(ng/ml)	658 ± 241	915 ± 352	<0.001

Data are presented as the mean ± standard deviation, number (percentage), or median (interquartile range).

**FIGURE 5:**
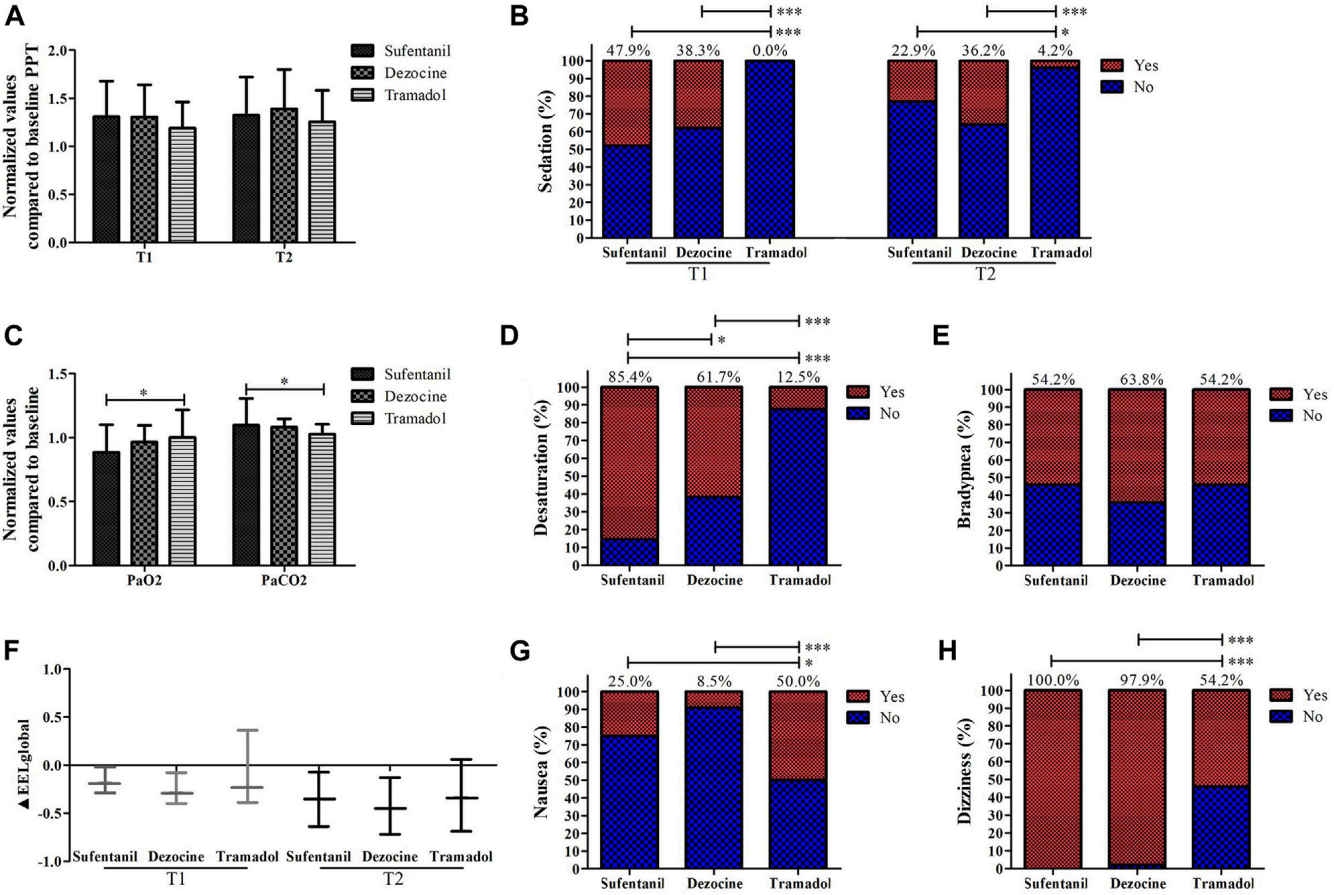
Comparisons of the values of analgesic effects **(A)**, sedation **(B)**, PaO_2_ and PaCO_2_ **(C)**, the incidences of desaturation **(D)**, bradypnea **(E)**, ΔEELI **(F)**; nausea **(G)** and dizziness **(H)** in the morning group for patients receiving sufentanil, dezocine and tramadol. (T1, onset time of analgesic effect; T2, time of peak concentration; *p < 0.05, **p < 0.01 and ***p < 0.001 compared with the afternoon group).

Comparisons of analgesic effects and other side effects in the morning group among different analgesics are shown in Figure 5, and no significant differences existed in demographic and baseline characteristics among the sufentanil, dezocine, and tramadol groups (Supplementary Table S1). No significant difference was found in normalized values compared with baseline PPT among the different analgesics. The incidences of sedation, dizziness, and desaturation in the tramadol group were lower than those in the other groups. The normalized value compared to the baseline PaO_2_ in the tramadol group was higher than that of the sufentanil group, and the normalized value compared to the baseline PaCO_2_ was significantly lower in the tramadol group. However, the incidence of nausea was significantly higher in the tramadol group than in the sufentanil and dezocine groups.

## Discussion

In contrast to previous studies on animals or small numbers of volunteers, which focused on the differences between day and night administration of analgesics, this study included patients undergoing elective surgery under general anesthesia and observed the difference in the analgesic effect of intravenous equivalent doses of tramadol, dezocine, and sufentanil in the morning and afternoon. Our study also investigated differences in cardiovascular reactions, respiratory depression, nausea, and vomiting at different time points. Therefore, this study has direct guiding significance in clinical practice. In this study, we found that the intravenous administration of sufentanil, dezocine, and tramadol in the morning produced a better analgesic effect than in the afternoon. In terms of side effects, tramadol is more prone to nausea and vomiting in the morning, dezocine is more prone to hypoxemia and hypercapnia in the morning, and sufentanil is more prone to sedation in the afternoon. In addition, when opioid analgesics with different mechanisms are used in the morning, there are significant differences in the occurrence of adverse reactions under similar analgesic effects.

Previous studies have explored the chronopharmacology of μ-receptor-activated analgesics in different ways. Some studies have found that the analgesic effect in the dark phase is greater than in the light phase after morphine injection in mice, and this difference is related to the degree of μ-receptor expression in the brain (Yoshida et al., 2003; Yu et al., 2015). In this study, we determined that intravenous sufentanil administration in the morning produces a better analgesic effect. Pharmacology and pharmacokinetics may affect the time-related pharmacodynamics (Dallmann et al., 2014). In this study, we found that there was no difference in the blood concentration of sufentanil in the morning and afternoon when the blood concentration of sufentanil reached its peak, which indicated that the time difference in the analgesic effect of sufentanil might be due to pharmacological rather than pharmacokinetic differences. It is known that sufentanil mainly activates the μ-receptor, hyperpolarizes the postsynaptic membrane through the G-protein coupling mechanism, and prevents the conduction of pain impulses, thus exerting an analgesic effect (Pasternak, 1993; Zöllner and Stein, 2007; Zöllner and Schäfer, 2008). A previous study found that the expression of μ-receptors in the periaqueductal gray at 14:00 p.m. was significantly higher than that at 8:00 a.m. in mouse models (Takada et al., 2013). In contrast, we speculate that in humans, the level of μ-receptors in the periaqueductal gray is higher in the morning than in the afternoon. Therefore, the difference in sufentanil efficacy between the morning and afternoon may be due to the circadian rhythm of μ-receptor expression. In addition, we found that the incidence of the sedative effect of sufentanil after 10 min of administration in the afternoon was higher than that in the morning. Sufentanil can combine with the μ-receptor of the central nervous system to exert analgesic and sedative effects, and can also activate presynaptic receptors on GABA neurons to reduce central excitability (Zöllner and Stein, 2007; Trescot et al., 2008; Rocha et al., 2011; Huang et al., 2020). Animal studies have shown that the GABA neuronal response has a significant rhythm, and compared with ZT2, GABA neuronal reactivity at ZT6 is significantly lower (Wagner et al., 1997; Schellinger et al., 2022). In contrast, it can be speculated that GABA neuronal reactivity is stronger in the afternoon in humans. Therefore, stronger sedation with opioid application in the afternoon may be related to the time difference in GABA neuronal reactivity.

Dezocine is a κ-receptor agonist and μ-receptor agonist, which is an analgesic widely used in the perioperative period (Liu et al., 2014; Wang et al., 2018; Ye et al., 2021); however at present, there is no research to explore the chronopharmacology of this kind of analgesic. In this study, we found that the analgesic effect of dezocine in the morning was better than that in the afternoon; however, the time difference was not as obvious as that of sufentanil, which may be related to its different analgesic mechanisms. The blood test results showed that there was no significant difference in the blood concentration of dezocine at different times. Therefore, similar to sufentanil, we speculate that the reason for the time difference in the analgesic effect is related to the time difference in pharmacology. However, in the side effects we found that PaO_2_ decreased and PaCO_2_ increased significantly after dezocine administration in the afternoon compared to that in the morning. However, although the respiratory rate and pulmonary volume detected by EIT both decreased in the morning and afternoon, there was no difference in respiratory rate and EIT results between the different time durations. Thus, we speculated that dezocine administration in the afternoon might have a more significant inhibitory effect on the reactivity of the respiratory center. Activation of the μ-receptor can inhibit excitatory synaptic transmission in the respiratory center (Varga et al., 2020; Baertsch et al., 2021; Liu et al., 2021), while the expression of the μ-receptor may be lower in the afternoon than in the morning. Thus, the effect of antagonism of the μ-receptor for dezocine may also be weaker in the afternoon, so the application of dezocine in the afternoon can more strongly inhibit the respiratory network of the brain stem. However, this requires further validation in the future. Nevertheless, it is necessary consider this time difference in the clinical application of dezocine, and special attention should be paid to respiratory monitoring when using dezocine in the afternoon.

Tramadol is an inhibitor of serotonin and norepinephrine reuptake and has a μ-receptor excitatory effect (Grond and Sablotzki, 2004; Ogbemudia et al., 2022). One study had performed painful stimulation of the nasal mucosa of 18 healthy volunteers and found that the analgesic effect produced by oral tramadol at night was stronger (Hummel et al., 1995); in contrast to this study, we explored the role of daytime variation in the analgesic effect of intravenous tramadol, and the results showed that it was stronger in the morning, than in the afternoon. The degree and incidence of nausea and vomiting after tramadol administration in the morning were also higher than those in the afternoon. Blood sample tests show that the serum concentration of tramadol in the afternoon is significantly higher than that in the morning, CYP2D6 is the key metabolic enzyme of tramadol, and CYP2D6 converts tramadol into the active metabolite O-desmethyltramadol (M1) and plays a pharmacological role (Dean and Kane, 2012; Lassen et al., 2015; Miotto et al., 2017). However, previous studies have shown that CYP2D6 is less expressed in the afternoon than in the morning (Matsunaga et al., 2012); therefore it can be speculated that tramadol in the morning can be converted into an active metabolite by the CYP2D6 enzyme. This phenomenon contributes to the lower blood concentration of tramadol in the morning than in the afternoon, thus inducing stronger pharmacological effects in the morning.

The equivalent dose ratios of sufentanil, dezocine, and tramadol to morphine were 1:10000, 1:1 and 10:1 respectively (Pandit et al., 1985; Grond and Sablotzki, 2004; Fentanyl, 2012); thus, the doses of the three drugs in this study were equivalent. Based on the time comparison, we found that the analgesic effect was better in the morning. Therefore, we chose the morning to compare the comprehensive clinical effects of the three drugs. The results showed that there was no difference in the analgesic effect among the three drugs, which was consistent with the clinical equivalent dose. We found that PaO_2_ decreased and PaCO_2_ increased most significantly in the sufentanil group, and the incidence of hypoxic saturation was also significantly higher than in the other two groups, followed by dezocine; however, there was no significant difference in respiratory rate and EIT. This may be directly related to the pharmacological mechanisms of the three types of opioids. Respiratory depression is one of the most harmful side effects of opioids, and this finding might be helpful for choosing type and doses of analgesics when treatment is administered in the morning.

Regarding other side effects, we found that the incidence of nausea and vomiting caused by intravenous tramadol was the highest among the three drugs. The causes of nausea and vomiting caused by tramadol may be various, and their occurrence may be related to the activation of μ-receptors and 5-HT receptors in the chemical receptor trigger region (CTZ) of vomiting center in the fourth ventricle of the brain (Mallick-Searle and Fillman, 2017). Therefore, the incidence of adverse reactions of nausea and vomiting caused by intravenous tramadol was significantly higher than that caused by the other two opioids. When tramadol is administered in the morning, it is necessary to consider the occurrence of nausea and vomiting. In addition, compared to tramadol, sufentanil and dezocine produced significantly more sedative effects and dizziness. Therefore, when clinicians select drugs to make pain treatment plans, they should fully consider the differences in adverse reactions to opioids *via* different mechanisms.

Although the current research has yielded several interesting findings, it is necessary to consider the limitations of this research. First, while we observed the time-efficacy rhythms of opioid analgesics with different mechanisms at different time points, we only studied drugs commonly used in clinics at these times in the morning and afternoon, and further studies are needed to explore their complete circadian rhythm in other periods. Second, this study observed three representative opioids with different mechanisms; other opioids such as oxycodone, hydromorphone, and naborphine were not included. Because of the differences in drug structures, the specific time-pharmacodynamics of these analgesics in clinical practice remains unclear. Thirdly, this study didn’t record the subjects’ sleep time, which may be one confounding factor for difference of opioids’ pharmacological effects between the morning and afternoon, and its potential effects needs further exploration. In addition, the patients included in this study were selected according to relatively strict inclusion and exclusion criteria; thus, the role of daytime variation in these opioids’ clinical pharmaceutical effects for patients with various complications needs to be further explored. For example, some differences induced by daytime variations may be amplified or reduced by certain complications.

In this study, the clinical effects of these three commonly used opioid analgesics were comprehensively evaluated and compared using an experimental pain measurement method combined with real-time continuous monitoring technology and evaluated at different time points. Sufentanil, dezocine, and tramadol, which were intravenously administered in the morning had better analgesic effects, and the side effects of the three drugs also differed at different times. In addition, the side effects of the three drugs used during the same period were significant. Therefore, when clinicians use these three kinds of opioids in their daily clinical practice, it is necessary to formulate individualized treatment plans according to different times and patient conditions to obtain maximum analgesic effect and minimum side effects.

## Data Availability

The raw data supporting the conclusion of this article will be made available by the authors, without undue reservation.

## Supplementary material

SUPPLEMENTARY FIGURE S1 Comparisons of the mean arterial pressure (1) and heart rate (2) during test procedure between the morning and afternoon groups for patients receiving sufentanil **(A)**, dezocine **(B)** and tramadol **(C)**.

## Abbreviations

ABGs, arterial blood gas analysis; EIT, electrical Impedance Tomography; EELI, end-expiratory lung impedance; ELISA, enzyme-linked immunosorbent assay; HPLC, high-performance liquid chromatography; MOAA/S, Modified Observer’s Assessment of Alertness/Sedation; NRS, numeric rating scale; PPT, pressure pain threshold.
