# Supplementary material for: Role of daytime variation in pharmaceutical effects of sufentanil, dezocine, and tramadol: A matched observational study
Source: Front Pharmacol. 2022 Sep 16;13:993506. doi: 10.3389/fphar.2022.993506 (PMC9523536; doi:10.3389/fphar.2022.993506)
Supplement: Supplementary file 1 [file Table1.docx]

Table S1. Demographic and baseline characteristics for subjects in the morning group among different analgesics.

|  | Sufentanil | Dezocine | Tramadol | *P* |
| --- | --- | --- | --- | --- |
| Age (year) | 44.5±10.6 | 41.6±9.4 | 42.4±10.9 | 0.376 |
| Sex (Male) | 14(29.2%) | 9(19.1%) | 10(20.8%) | 0.461 |
| Weight (kg) | 62±9 | 62±9 | 60±8 | 0.280 |
| Height (cm) | 161±7 | 161±8 | 161±6 | 0.864 |
| BMI (kg/m^2^) | 23.9±2.8 | 23.8±2.8 | 23.0±2.6 | 0.247 |
| HEI | 4.0(1.0~8.2) | 4.0(2.0~7.0) | 5.0(3.0~7.0) | 0.861 |
| MAP (mmHg) | 99±13 | 97±10 | 94±11 | 0.159 |
| HR (bpm) | 76±11 | 76±12 | 75±8 | 0.673 |
| RR (bpm) | 15.3±3.0 | 15.6±3.7 | 16.5±4.4 | 0.891 |
| S_P_O2 (%) | 98.5±1.5 | 98.6±1.5 | 98.6±1.4 | 0.237 |

Data are presented as mean ± standard deviation, number (percentage) or median (interquartile range).
